# The mitomiR/Bcl-2 axis affects mitochondrial function and autophagic vacuole formation in senescent endothelial cells

**DOI:** 10.18632/aging.101591

**Published:** 2018-10-21

**Authors:** Angelica Giuliani, Ilenia Cirilli, Francesco Prattichizzo, Emanuela Mensà, Gianluca Fulgenzi, Jacopo Sabbatinelli, Laura Graciotti, Fabiola Olivieri, Antonio Domenico Procopio, Luca Tiano, Maria Rita Rippo

**Affiliations:** 1 Department of Clinical and Molecular Sciences (DISCLIMO), Università Politecnica delle Marche, Ancona, Italy; 2 Department of Life and Environmental Sciences, Università Politecnica delle Marche, Ancona, Italy; 3 IRCCS MultiMedica, Milano, Italy; 4 Neural Development Section, Mouse Cancer Genetics Program, CCR, NCI, Frederick, MD 1702, USA; 5 Center of Clinical Pathology and Innovative Therapy, INRCA-IRCCS National Institute, Ancona, Italy

**Keywords:** mitomiRs, Bcl-2, apoptosis, autophagy, senescence

## Abstract

During senescence, cells undergo distinctive biochemical and morphological changes and become dysfunctional. MiRNAs are involved in the senescence process and specific miRNAs can localize to mitochondria (mitomiRs). We hypothesized that part of the typical alterations of senescence may depends on mitomiRs deregulation. Therefore, we thoroughly explored the phenotype of human endothelial cells undergoing replicative senescence (sHUVECs) and observed elongated/branched mitochondria, accumulation of autophagic vacuoles (AVs), increased ROS and IL-1β production and reduced expression of Bcl-2 compared to younger cells (yHUVECs). Despite these pro-apoptotic features, sHUVECs are more resistant to serum deprivation, conceivably due to development of pro-survival strategies such as upregulation of Bcl-xL and Survivin. We demonstrate that mitomiR-181a, -34a, and -146a, are overexpressed and localize to mitochondria in sHUVECs compared with yHUVECs and that they: i) down-regulate Bcl-2, ii) induce permeability transition pore opening and activation of caspase-1 and 3, iii) affect sensitivity to apoptosis and iv) promote the conversion of LC3-I to LC3-II. Overall, we document for the first time that some mitomiRs can act as mediators of the multiple but functionally linked biochemical and morphological changes that characterize aging cells and that they can promote different cellular outcomes according to the senescence status of the cell.

## Introduction

Senescent cells (SC)s are emerging as major drivers of aging and age-related diseases (ARD)s [1]. SCs are characterized by dysfunctional mitochondria, which are partly responsible of their detrimental effect on tissue homeostasis [2]. The balance between mitochondrial fission and fusion, which is critical for the functionality of the mitochondrial network, is also altered in SCs [3]. This imbalance leads to accumulation of damaged and dysfunctional mitochondria which, in turn, fail to be eliminated due to defective autophagy [4]. Importantly, damaged or dysfunctional mitochondria, by producing reactive oxygen species (ROS) and releasing other mediators like mitochondrial DNA (mtDNA) and oxidized mtDNA [5,6], can promote a distinctive sterile, chronic low-grade inflammatory status that has been designated inflamm-aging and contribute to its maintenance. Indeed, efficient autophagy promotes healthy aging [7].

MicroRNAs (miRNAs or miRs) are small non-coding RNAs. They work as fine tuners of protein expression: by binding to specific complemental seed sequences, they mediate degradation, repress translation or, in some cases, stabilize their target mRNAs. One miRNA can target multiple mRNAs and, conversely, multiple miRNAs can target a single mRNA. MiRNAs play significant roles in all the regulatory mechanisms of the cells, including senescence [8,9]. Notably, a small pool of nuclear-coded miRNAs is found within mitochondria (mitomiRs) [10]. Although their role is still largely unknown, some data suggest that they may modulate the expression of functional mitochondrial proteins [11–13] and that they can translationally regulate mitochondria-encoded proteins [14,15].

We have previously advanced the hypothesis that some of the several miRNAs that are modulated during aging and cellular senescence (senescence-associated miRs, SA-miRs) and are directly involved in SC dysfunction are mitomiRs (SA-mitomiRs). We have also shown that a small group of SA-mitomiRs can target Bcl-2 family members and that the principal member of the family, Bcl-2, is targeted by the largest number of validated as well as putative regulating mitomiRs. These data suggested to us that SA-mitomiRs could affect SC mitochondrial function by regulating Bcl-2 expression [16,17]. Bcl-2 is a validated target of miR-181a, -34a, and -146a and is modulated in different cellular systems and ARDs [18–20]. Notably, its physiological roles include not only the inhibition of apoptosis, but also of autophagy and ROS production [21], the promotion of mitochondrial fusion in non-apoptotic cells [22], and the inhibition of NLRP1, an inflammasome core component [23].

Here, we set out to test our hypothesis that mitomiRs play a role in SCs dysfunction using a prototypical model of cell aging, primary human umbilical vein endothelial cells (HUVECs) undergoing replicative senescence [24,25]. We demonstrated that miR-181a, miR-34a, and miR-146a, three key mitomiRs, are increased in senescent HUVECs (sHUVECs) and reduce Bcl-2 expression. Then, since Bcl-2 modulation could affect both autophagy and apoptosis, we explored if sHUVEC phenotype could be attributed to mitomiRs deregulation. We finally suggest a hypothesis based on their strong ability to adapt to and withstand adverse conditions.

## RESULTS

### Senescent HUVECs show altered mitochondrial activity and morphology

**Figure 1:**
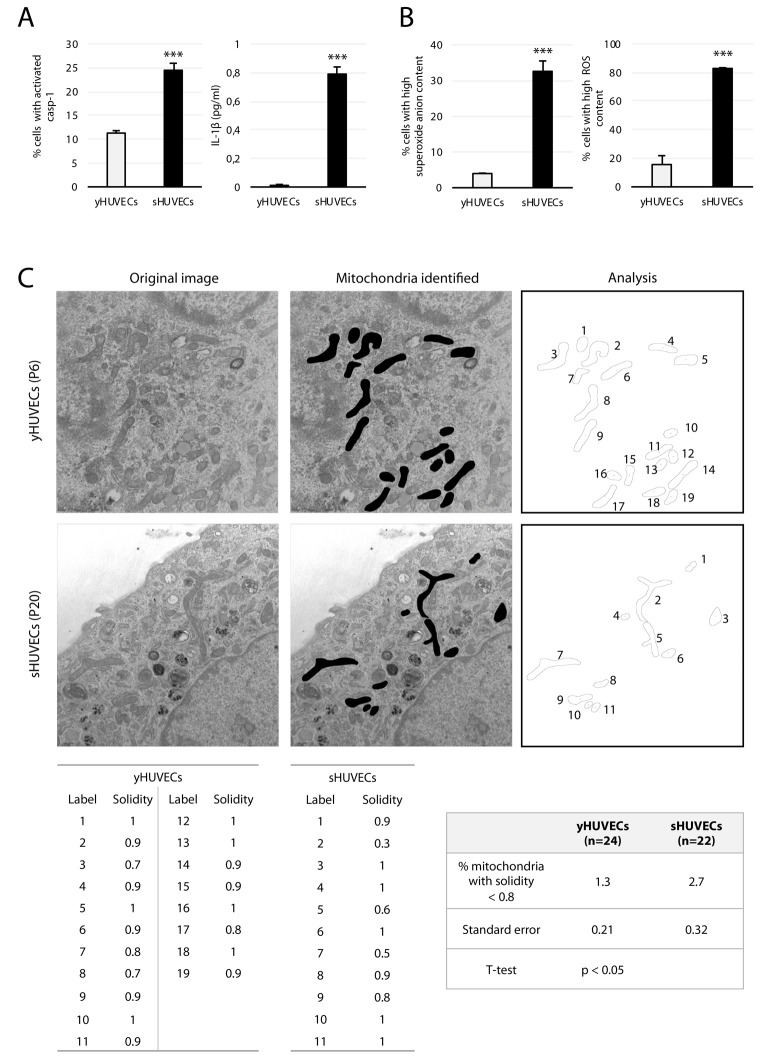
**Biochemical features of sHUVECs.** (**A**) Cytofluorimetry analysis of the percentage of cells with activated casp-1 (left) and of IL-1β concentration (pg/ml) in the culture medium (right) in yHUVECs and sHUVECs. (**B**) Percentage of HUVECs with high levels of anion superoxide (left) and ROS (right) production evaluated by flow cytometry; (**C**) Representative TEM image of mitochondria from a yHUVEC and a sHUVEC, original magnification 19 K. Images analyzed in ImageJ for shape descriptors. A solidity threshold of 0.8 was adopted to select for rougher and branched mitochondria and calculate their percentage out of the total number of mitochondria found in the image (n = number of cells analyzed at a magnification of 13 K to 19 K). *** t-test, p < 0.001.

In a previous work we have shown that HUVECs undergoing replicative senescence show all biochemical features typical of SCs: among these, positivity for senescence-associated β galactosidase (SA β-Gal) when growth has arrested [9,26] (Suppl. Fig. 1A) and upregulation of p16(Ink4a), a major marker of SCs (Suppl. Fig. 1B). Here we also measured the release of IL-1β, which derives from cleavage of its pro-IL-1β precursor via caspase (casp)-1, and assessed casp-1 activation. Casp-1 activity and IL-1β were both significantly higher in senescent than in young HUVECs (yHUVECs) (Fig. 1A).

Since SCs are suggested to carry a high burden of oxidative stress and mitochondrial alterations [27,28], we analyzed the percentage of sHUVECs showing high levels of mitochondrial superoxide anion and cytosolic ROS, and compared these values to those measured in young cells. As expected, these species were strongly overproduced in sHUVECs (Fig. 1B).

In addition, we used TEM to measure mitochondrial perimeter and solidity, the two key morphological parameters that predict fission and fusion events. The perimeter is a mitochondrion’s border, whereas solidity is a measure of its shape complexity (a tortuous or branched mitochondrion has a longer perimeter and is less solid than a compact mitochondrion). Perimeter positively correlates with a future fission event and solidity with a future fusion event [29]. TEM analysis demonstrated significant morphological changes, i.e. tortuous, branched and less solid mitochondria in sHUVECs compared with yHUVECs (Fig. 1C).

**Figure 2:**
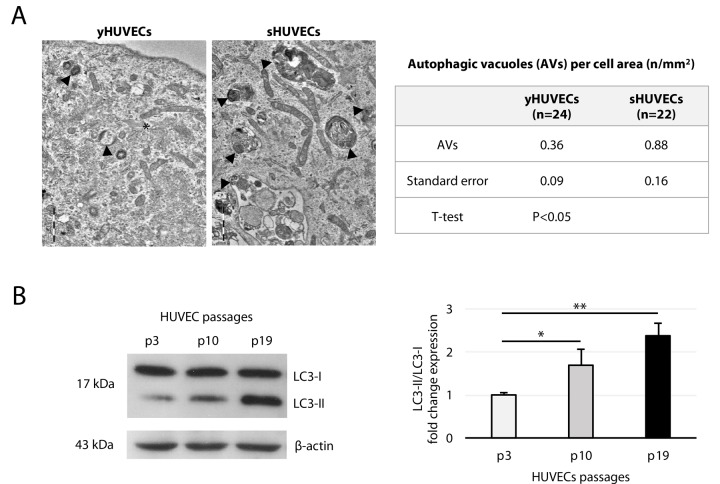
**Autophagic vacuoles (AV)s and LC3 expression in yHUVECs and sHUVECs.** (**A**) TEM images of AVs (arrowheads) in yHUVECs and sHUVECs (left); AVs were quantified per cell area (right), n = number of cells analyzed. Asterisk near a Weibel Palade body. Magnification, 19 K. (**B**) Western blot and densitometric analysis of LC3-II/LC3-I ratio fold change to P3 (yHUVECs) during replicative senescence. Data were normalized to β-actin. Data are mean ± SD of three independent experiments. * t-test p < 0.05; ** t-test p < 0.01.

We also assessed the young and senescent HUVECs autophagic state analyzing i) the number of autophagic vacuoles (AVs) using TEM and ii) microtubule-associated protein 1A/1B-light chain 3 (LC3) expression [30]. TEM demonstrated a larger number of AVs in sHUVECs compared with yHUVECs (Fig. 2A, left panel, arrows). Semi-quantitative analysis indicated that AVs / μm^2^ were more than twice more numerous in sHUVECs than in yHUVECs (Fig. 2A, right panel). Moreover, progressively and significantly increased LC3-I to LC3-II conversion was seen in aging HUVECs (Fig. 2B).

Overall, these data indicate that sHUVECs are characterized by a high burden of oxidative stress and by an altered mitochondrial morphology, along with an increased number of AVs.

### sHUVECs are more resistant to apoptosis than yHUVECs despite Bcl-2 downregulation

**Figure 3:**
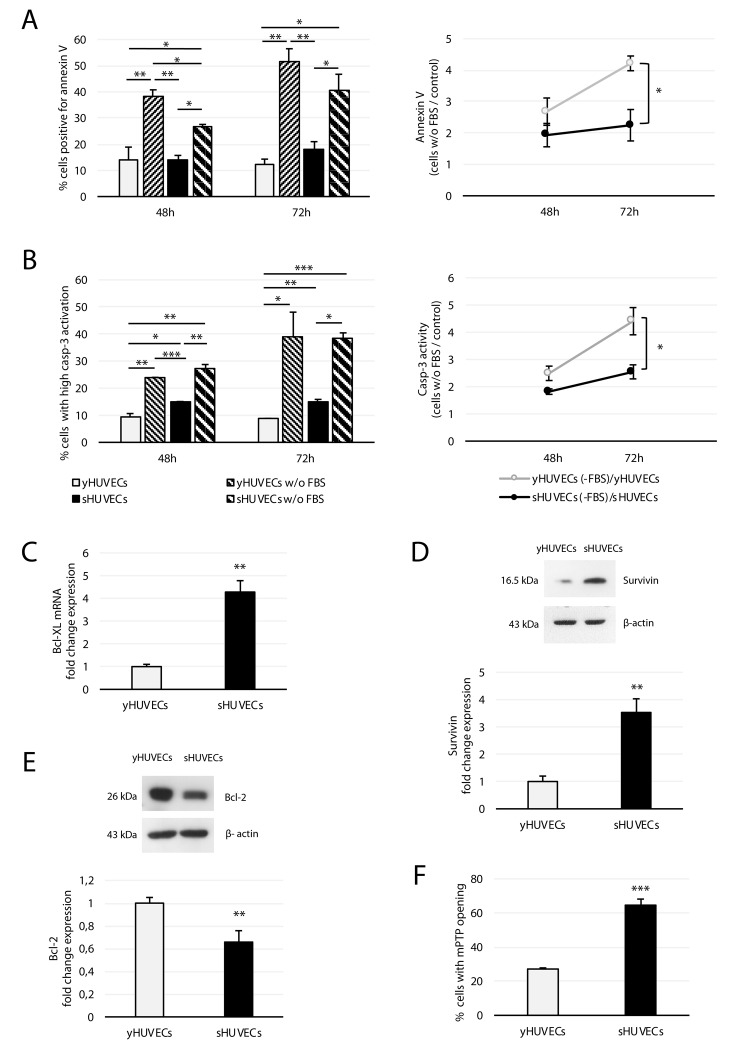
**Effect of FBS deprivation on young and senescent HUVECs***.* yHUVECs and sHUVECs were cultured for 48 (48h) or 72 (72h) hours with or without (w/o) FBS. Annexin V positivity and casp-3 activation were analyzed by flow cytometry. (**A**) Percentage of annexin V-positive cells (left) and ratio of annexin V-positive apoptotic cells among yHUVECs and sHUVECs w/o FBS to their control (with FBS) (right). (**B**) Percentage of cells with active casp-3 (left); ratio of yHUVECs or sHUVECs w/o FBS with activated casp-3 to control cells (right). (**C**) Bcl-xL mRNA fold change in yHUVECs and sHUVECs. (**D**) Western blot and densitometric analysis of Survivin expression in yHUVECs and sHUVECs. (**E**) Western blot and densitometric analysis of Bcl-2 in yHUVECs and sHUVECs. (**F**) Percentage of HUVECs showing mPTP opening. Protein expression values are reported as Bcl-2 and Survivin fold change in sHUVECs vs yHUVECs. Data are normalized to β-actin protein. Data are mean ± SD of three independent experiments. * t -test p < 0.05, ** t-test p < 0.01, *** t-test p < 0.001.

Since resistance to apoptosis has been proposed as a major mechanism leading to accumulation of senescent cells with aging [31,32], we compared the resistance of young and senescent cells to death using serum deprivation as a stress stimulus for 48 and 72 h. At 48 h the percentage of spontaneous apoptotic cells was similar in young and senescent HUVECs, whereas at 72 h sHUVECs showed a slightly greater susceptibility to undergo apoptosis (Fig. 3A, left panel), in line with previous findings [33]. However, sHUVECs seemed to be more resistant to serum deprivation. In fact, only 27% of sHUVECs vs 38% of yHUVECs were positive for annexin V at 48 h, and only 40% of sHUVECs vs with 52% of yHUVECs were positive for annexin V at 72 h (Fig. 3A, left panel). The diagrams reported in Fig. 3A (right-side panel) demonstrate that the sHUVECs and yHUVECs subjected to serum deprivation showing positivity for annexin V were respectively two and four times more numerous compared with control cells. Casp-3 was activated by serum deprivation in both sets of HUVECs (Fig. 3B). Interestingly, although sHUVECs displayed higher spontaneous casp-3 activity (Fig. 3B, left panel), its activation after serum deprivation was higher in yHUVECs, in line with the results obtained with annexin V in terms of fold increase vs baseline (Fig. 3B right-side panel).

To gain further insight into the mechanisms underlying the resistance to apoptosis we observed in sHUVECs, we analyzed the expression of the anti-apoptotic proteins Bcl-2, Bcl-xL and survivin. Bcl-2 family members control critical steps in the commitment to apoptosis by regulating mitochondrial membrane permeabilization [34]; in particular, Bcl-2 regulates mPTP opening, which is believed to relate directly to ROS generation [35].

RT-PCR and Western blot analysis respectively showed that Bcl-xL and Survivin are upregulated in sHUVECs (Fig. 3C and D). However, Bcl-2 is downregulated in sHUVECs both at the protein (Fig. 3E) and the mRNA level (fold change = 0.33, p <0.01), consistent with previous reports [33]. Accordingly, a large proportion of cultured sHUVECs presents mPTP opening (Fig. 3F). Therefore, despite Bcl-2 downregulation and mPTP opening, sHUVECs resistance to serum deprivation seems to be conferred by other anti-apoptotic proteins, such as Bcl-xL and Survivin.

### MitomiR-181a, -34a, and -146a are upregulated in sHUVECs and regulate Bcl-2 expression

**Figure 4:**
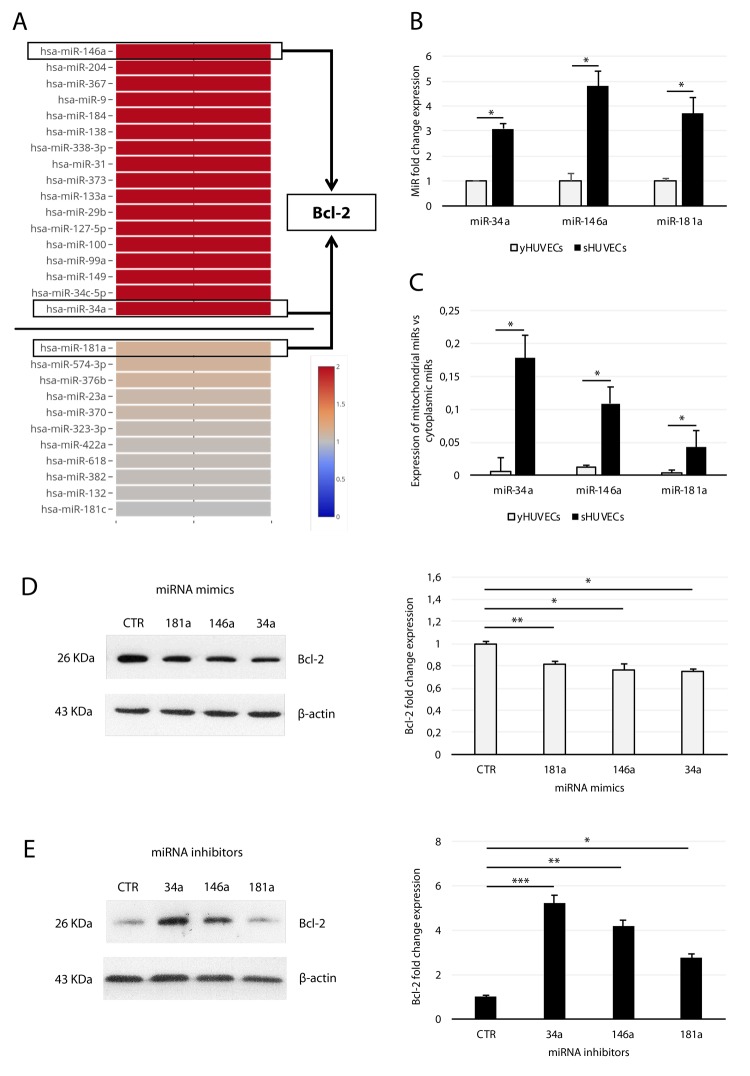
**Analysis of miR-34a, -146a, and -181a in sHUVECs and their effect on Bcl-2 expression.** (**A**) Heatmap showing the expression of selected miRNAs in sHUVECs compared to yHUVECs. Expression level of each miRNA is depicted according to the color scale. Adapted from Olivieri et al. [26]. (**B**) Fold increase of miR-34a, -146a, and -181a in senescent and young HUVECs. (**C**) Ratio of miR-34a, -146a, and -181a expression in the isolated mitochondrial fraction to the cytoplasmic fraction in yHUVECs and sHUVECs. (**D**) Western blot and densitometric analysis of Bcl-2 expression in yHUVECs transfected with miRNA mimics (miR-34a, miR-146a, and miR-181a) and negative miRNA mimic control (CTR). (**E**) Western blot and densitometric analysis of Bcl-2 expression in sHUVECs transfected with miRNA inhibitors (miR-34a, miR-146a, and miR-181a) and negative miRNA inhibitor control (CTR). Protein expression values are reported as Bcl-2 fold change in sHUVECs vs yHUVECs. Data are normalized to β-actin protein expression. Data are mean ± SD of three independent experiments. * t-test p < 0.05, ** t-test p < 0.01, *** t-test p < 0.001 vs CTR.

In our previous work, we advanced the hypothesis that several miRs, which according to profiling data are modulated in sHUVECs (Fig. 4A) [26], were mitomiRs and affected mitochondrial function in SCs by acting on Bcl-2 family members [16,20,36]. To test this hypothesis, we first validated the expression of miR-34a, -146a, and -181a by qRT-PCR. This analysis demonstrated that they are all significantly upregulated in sHUVECs compared with yHUVECs (Fig. 4B). Furthermore, we assessed the presence of these mitomiRs within isolated mitochondria. Analysis of these data indicated that the ratio of miRs residing in mitochondria to those residing in cytosol was higher in sHUVECs than in yHUVECs (Fig. 4C). The purity of isolated mitochondria was assessed by analyzing the expression of the mitochondrial Voltage-Dependent Anion Channel (VDAC) and of the nuclear lamin A/C proteins as well as of the miR-370 (not classified as a mitomiR) in isolated mitochondria (Suppl. Fig. 2). These data suggest a shift towards a mitochondrial subcellular localization of these three miRNAs in sHUVECs.

To demonstrate a relationship between Bcl-2 and miR-181a, -34a, and -146a, we forced their expression with miRNA mimics and examined Bcl-2 modulation in yHUVECs. Separate transfection with each miRNA resulted in partial but significant downregulation of Bcl-2 protein. Specifically, the miR-34a mimic proved to be the most efficient Bcl-2 modulator (Fig. 4D). Accordingly, the expression of Bcl-2 in sHUVECs was rescued by transfection with the specific inhibitors (antagomiRs) of the three miRNAs (Fig. 4E), with miR-34a inhibitor exerting the greatest effect.

Therefore, up-regulated mitomiR-181a, -34a, and -146a localize in the mitochondrial compartment and downregulate Bcl-2 in sHUVECs.

### MitomiR-181a, -34a, and -146a coordinately regulate LC3-I to LC3-II conversion in young and senescent cells

Based on our finding that Bcl-2 expression is negatively modulated by mitomiR-181a, -34a, and -146a, and because of its involvement in autophagy inhibition [37], we investigated the potential involvement of these mitomiRs in AV formation.

**Figure 5:**
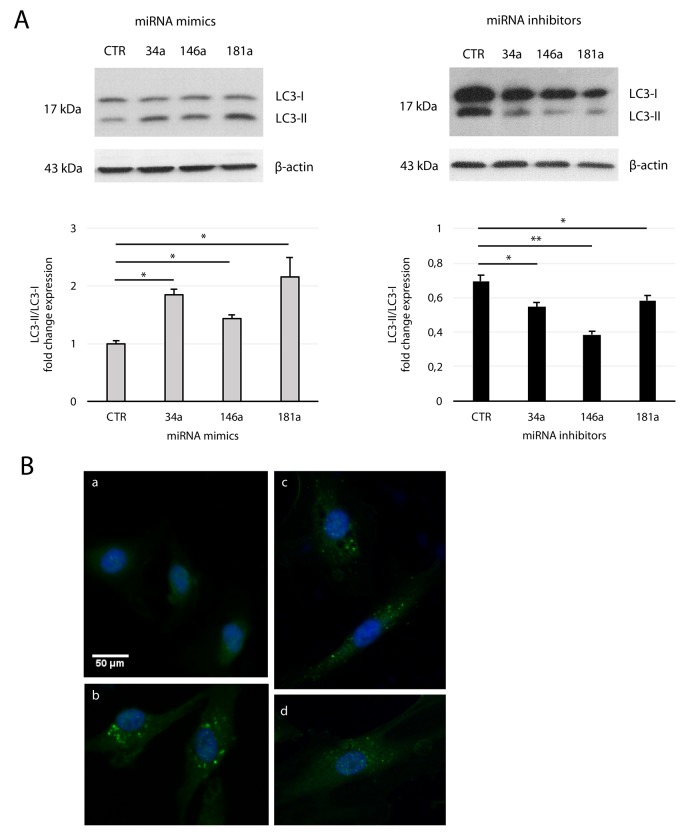
**Effect of mitomiRs on LC3 regulation in HUVECs.** (**A**) Western blot and densitometric analysis of LC3-II/LC3-I ratio fold change in yHUVECs (left) and sHUVECs (right) transfected with miRNA (miR-34a, miR-146a and miR-181a) mimics and inhibitors, respectively. Data were normalized to β-actin. Data are mean ± SD of three independent experiments. * t-test p < 0.05; ** t-test p < 0.01. (**B**) Representative immunofluorescence of yHUVECs transfected with CTR (**a**) or miRNA mimics miR-34a (**b**), miR-146a (**c**), miR-181a (**d**), using LC3 antibody (green fluorescence). Nuclear DNA was labelled with HOECHST (blue).

Transfection of yHUVECs with miRNA mimics enhanced the conversion of LC3-I to LC3-II, with a fold change expression of LC3-II vs LC3-I similar to that observed on sHUVECs (Fig. 5A and Fig. 2B), while miRNA inhibitors reverted this process in sHUVECs. These results were confirmed by immunofluorescence staining of LC3-II on miRNA mimics-transfected yHUVECs (Fig. 5B). Overall, these data suggest that SA-mitomiR-181a, -34a, and -146a foster AV formation in sHUVECs.

### MitomiR-181a, -34a, and -146a induce mPTP opening, apoptosis, and casp-1 activation in yHUVECs

**Figure 6:**
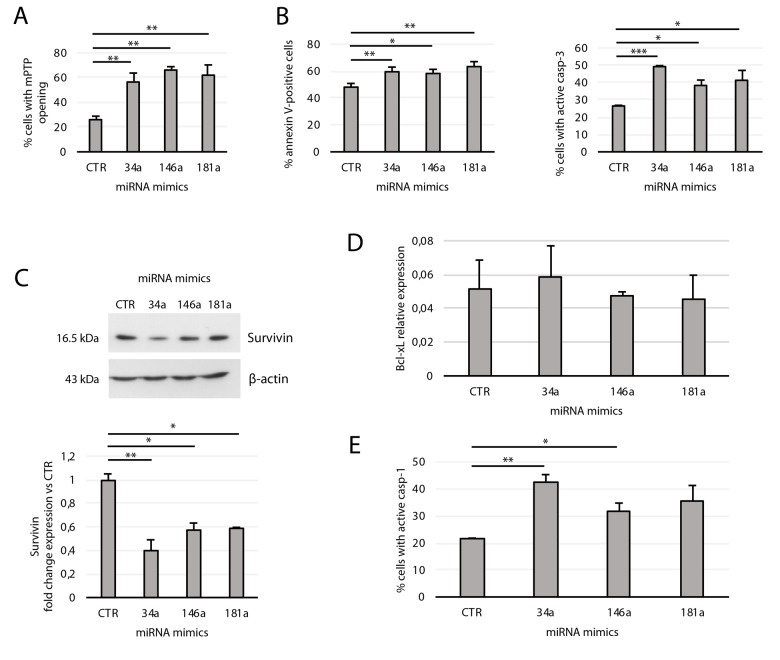
**Effects of transfection of the miRNA mimics 34a, 146a, and 181a on yHUVECs.** yHUVECs were incubated for 24 h with the miRNA mimics (miR-34a, -146a, and -181a) or with a miRNA mimic negative control (CTR) before analysis*.* The percentage of transfected yHUVECs showing (**A**) mPTP opening, (**B**) annexin V positivity and active casp-3, and (E) casp-1 were evaluated by flow cytometry. (**C**) Western blot and densitometric analysis of Survivin expression in transfected yHUVECs. Survivin expression is reported as fold change in miRNA mimic-transfected cells vs CTR. (**D**) Bcl-xL mRNA relative expression in transfected yHUVECs. Data are mean ± SD of three independent experiments. * t-test p < 0.05, ** t-test p < 0.01, *** t-test p < 0.001.

Given the effect of miR-181a, -34a, and -146a mimics in downregulating Bcl-2, we tested their effect on mPTP opening and apoptosis. Forced expression of each of the three miRNAs in young transfected cells strongly affected mPTP opening (Fig. 6A). Accordingly, cytofluorimetric analysis of young transfectants showed increased annexin V positivity and casp-3 activation, with miR-34a showing the strongest ability to activate casp-3 (Fig. 6B). Since this higher efficiency could be due to its potential, but indirect, inhibitory effect on Survivin [38], and considering that Survivin regulates negatively programmed cell death by acting downstream on mitochondria and mPTP opening [39], we measured its expression in transfected cells. We found that Survivin expression was reduced by all three mitomiRs, with miR-34a again exerting the strongest effect (Fig. 6C). As expected, miRNA mimics did not modulate Bcl-xL expression since it is not a target of these mitomiRs (Fig. 6D).

We also examined the effect of miR-181a, miR-34a, and miR-146a transfection in promoting casp-1 activation. Notably, Bcl-2 downregulation can induce casp-1 activation [40]; furthermore, mitochondrial dysfunction and oxidative stress can induce and potentiate inflammatory responses [5]. As shown in Figure 6E, miR-146a and miR-34a are both strong inducers of casp-1 activity. Collectively, these data indicate that each of the three mitomiRs induces mitochondrial dysfunction and promotes apoptosis in yHUVECs. Furthermore, miR-34a and miR-146a also induce the pro-inflammatory caspase-1 activation.

## DISCUSSION

Mitochondria are emerging as major determinants of SC pathogenicity and of organismal aging [41]. Various miRNAs have been demonstrated to influence mitochondrial function. Those localized to mitochondria have been designated as “mitomiRs” [10]. Since mitomiRs are a recent discovery, their role in the aging process is largely unknown. Among the miRNAs previously reported to be involved in the senescence processes, we previously identified three mitomiRs (miR-181a, miR-34a, and miR-146a) as candidate regulators of mitochondrial activity in SCs; we called them “SA-mitomiRs” [16] and suggested that they could promote mitochondrial dysfunction via Bcl-2 downregulation. Indeed, several studies have documented that these miRNAs can target the mitochondrial protein Bcl-2 in different models [19,20,42] and, furthermore, that miR-34a/miR-181a-Bcl-2 axis is sensitive to caloric restriction and its regulation prevents neuronal loss during aging [43]. Accordingly, IPA (Ingenuity Pathway Analysis) analysis suggested that mitomiRs -181a, -34a, and -146a may be involved in the senescence process and ARDs (cancer, skeletal and muscle disorders, cardiovascular, metabolic and neurological diseases) development [16]. In a previous study we had investigated changes in miRNA profiles in young and senescent HUVECs and the role of miR-146a as a modulator of inflamm-aging [26].

In the present study, we demonstrate that:

1 - specific nuclear-coded, SA-mitomiRs (miR-181a, miR-34a, and miR-146a) can be localized to and enriched in sHUVECs mitochondria and that they can downregulate the expression of their specific target, Bcl-2;

2 - forced expression of these SA-mitomiRs in young cells induces mitochondrial dysfunction, activates pro-apoptotic and pro-inflammatory caspases and promotes apoptosis;

3 - SCs are resistant to stress-induced death despite the modulation of mitomiRs and their target Bcl-2, possibly due to a concomitant increase in the expression of other anti-apoptotic proteins;

4 - SCs are characterized by an increase in autophagic vacuole formation that is attributable, at least in part, to the mitomiRs-Bcl-2 axis.

Bcl-2 modulation and its role in SCs have been the subject of some conflicting reports [33,44]. The different findings may be due to the different senescence inducers (*e.g.* replicative senescence, ultraviolet light, or ROS) used in these studies. Of note, some Bcl-2 family members have been identified as targets of senolytic drugs, molecules that specifically induce death in senescence-resistant cells. Zhu and co-workers have demonstrated that several molecules targeting Bcl-xL, including the senolytic drug navitoclax, exert a pro-apoptotic effect on SCs, as well as the direct Bcl-xL silencing through siRNA transfection [44–46]. Our data show that miR-181a, -34a, and -146a can induce downregulation of Bcl-2, but not of Bcl-xL, which is, on the contrary, more expressed in sHUVECs than in yHUVECs. Accordingly, Bcl-xL is neither a putative nor a validated target of these mitomiRs, but is regulated by let-7b [47], which is not regulated during HUVEC senescence [26]. Furthermore, we show that sHUVECs express high levels of the antiapoptotic molecule Survivin. Interestingly, Survivin siRNA had not yet been screened to develop senolytic molecules [44–46]. However, Al-Khalaf et al. reported that its inhibition with flavopiridol or specific shRNAs increases the apoptotic response of senescent fibroblast to various stressors [48]. As a result, sHUVECs are more resistant to apoptosis despite i) Bcl-2 downregulation and ii) exhibition of several characteristics of dysfunctional cells in which the early stages of programmed cell death have been activated, like ROS production, a significant increase in mPTP opening, and slight casp-3 activity, all findings that may be the result of Bcl-2 downregulation. Interestingly, our results are in line with the recent observation that SCs selectively release small extracellular vesicles loaded with miRNAs exerting antiapoptotic effects [49].

Unlike sHUVECs, yHUVECs, who have not programmed anti-apoptotic mechanisms, undergo apoptosis when miR-181a, -34a, and -146a are overexpressed. These results are in accordance with current knowledge suggesting that activated SCAPs (senescent cell anti-apoptotic pathways) can be targeted to promote SC clearance [50]. However, different results have been obtained according to the trigger used to promote apoptosis and the cell type involved. Indeed, sHUVECs are less prone to UV-induced cell death [51], while they appear more susceptible to death receptor-induced apoptosis [52]. Current research is progressively disentangling the cellular and context specificity of SCAPs activation [50].

The mitomiRs up-regulated in sHUVECs can induce mPTP opening in yHUVECs, even though by unknown mechanism. Accordingly, a recent report has recently shown that miR-7, another mitomiR involved in inflamm-aging and presumably in mitochondrial dysfunction during aging [15,17], can regulate the function of mPTP in human neuroblastoma cells and mouse primary neurons by targeting VDAC1, a component of the mitochondrial pore complex [53]. We also found that miR-34a, and to a lesser extent miR-146a can induce caspase-1 activation. Consistently, we detected substantial intrinsic casp-1 activity, which correlated with the release of its cleaved mature substrate, IL-1β, in sHUVECs. A decade ago, a leading team in the study of Bcl-2 and caspases demonstrated that Bcl-2 can inhibit activation of the proinflammatory molecule casp-1 by interacting with the NALP1 inflammasome complex [40]. Here we suggest that mitomiR-mediated Bcl-2 downregulation in aging cells could promote inflammasome activation.

Since Bcl-2 plays a key role in the autophagic process, we also explored autophagic vacuoles (AV)s formation in sHUVECs and in yHUVECs overexpressing the mitomiRs. We detected an increase in AV number in sHUVECs, paralleled by an increased activation of casp-3. Interestingly, Sirois and coworkers have demonstrated that casp-3 play a role in regulating AV release in endothelial cells [54]. Accordingly, forced expression of miR-181a, miR-34a, and miR-146a in young cells increased LC3-I to LC3-II conversion in sHUVECs, while specific antagomiRs exerted the opposite effect in sHUVECs.

**Figure 7:**
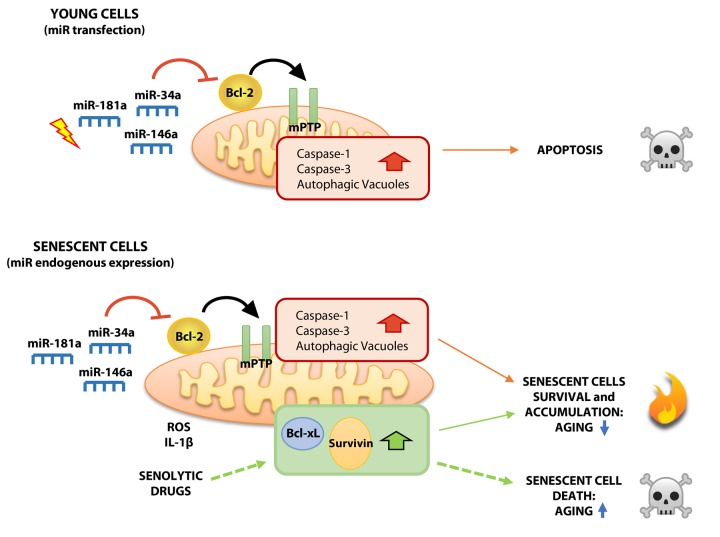
**Proposed model showing differential effects of mitoR-34a, -146a and -181a on young and senescent HUVECs.** Acute expression of mitomiRs in yHUVECs negatively modulates Bcl-2 expression, induces mPTP opening, caspase-1 and caspase-3 activation and autophagy. These biochemical and functional changes can be responsible of the pro-apoptotic effect exerted by mitomiRs. Conversely, although the progressive expression of miR-34a, miR-146a and miR-181a in sHUVECs during replicative senescence exerts biochemical changes similar to yHUVECs, they are more resistant to apoptosis, maybe due to the over-expression of other anti-apoptotic proteins (SCAPSs, i.e. Survivin and Bcl-xL) thus producing high amount of ROS and accumulating AVs. Overall, dysfunctional cells accumulate during aging, exerting detrimental effects on nearby cells and promoting inflammation. Senolytic drugs, by reducing over-expressed SCAPs, can ultimately kill dysfunctional sHUVECs.

Altogether, the present findings document two different effects of mitomiRs on young and senescent cells: their acute expression in yHUVECs exerts a pro-apoptotic effect, whereas their progressive and chronic increase in sHUVECs promotes cell dysfunction (Fig. 7). In the latter scenario, where at least the anti-apoptotic proteins Bcl-xL and Survivin are upregulated, we surmise that Bcl-2 downregulation, which is known to inhibit the early phases of autophagy [55], induces AV formation, leading to their accumulation and to increased LC3-I to LC3-II conversion in sHUVECs. This observation was also documented in young cells transfected with miRNA mimics. Although it is well established that autophagy is impaired in SCs [56], mitomiRs may nonetheless play a role in the process, enhancing its early phase by promoting autophagosome formation. It is conceivable that in SCs other mechanisms affect the next step, *i.e.* fusion of newly-formed autophagosomes and lysosomes. Although it is unclear whether the progressive AV accumulation in sHUVECs is due to their failure to fuse with lysosomes, its demonstration would raise the intriguing hypothesis that AVs serve as “dumps”, where severely damaged cell constituents are segregated from the working cell machinery. AVs could thus constitute a form of adaptation and survival to extreme conditions, like fused mitochondria. In fact, our sHUVECs showed tortuous and branched mitochondria, as reported in other studies. Such features have also been interpreted as reflecting hyperfusion or reduced fission, resulting in elongated mitochondria endowed with a greater ability to preserve energy and withstand adverse conditions [57]. Both interpretations are consistent with our TEM images. We are currently investigating this hypothesis.

Altogether, our data document for the first time that selected SA-mitomiRs can regulate mitochondrial function and autophagic behavior, suggesting a previously unrecognized link between epigenetic dysregulation and cell dysfunction during endothelial cell aging.

## MATERIALS AND METHODS

### Cell culture and growth conditions

Three different batches of pooled HUVECs were purchased from Clonetics (CC-2519, Lonza, Basel, Switzerland) and maintained in EGM-2 (CC-3162, Lonza) at 37 °C in a humidified atmosphere containing 5% CO_2_. Cells were seeded at a density of 5000/cm^2^; the medium was changed at 48–72 h intervals. Cultures were passaged when they reached confluence. Replicative senescence was assessed as described previously [25]. Cells were classified based on SA β-Gal activity into young (SA β-Gal < 5%) and senescent (SA β-Gal > 60%).

For serum deprivation experiments, cells were cultured in EGM-2 without fetal bovine serum for 48 or 72 h.

Senescence-associated expression of β-Gal activity was detected using Senescence Detection Kit (BioVision Inc., Milpitas, CA, USA). Briefly, non-confluent HUVECs cultured in 12-well plates were fixed for 15 min at room temperature, then washed twice in PBS. Cells were incubated overnight at 37°C with Staining Solution Mix (containing X-Gal). SA β-Gal was assessed by light microscopy. The percentage of positive cells was determined by counting at least 500 cells/well.

### Isolation of mitochondria

Mitochondrial isolation was performed with a specific kit based on Anti-TOM22 MicroBeads (Miltenyi Biotec Inc., Auburn, CA, USA), according to the manufacturer’s protocol. Briefly, 3x10^6^ cells were pelleted and lysed with lysis buffer containing protease inhibitors by 30 strokes with a 27 G needle. Next, the mitochondria were magnetically labeled by adding Anti-TOM22 MicroBeads to the lysate and incubated on a roller mixer for 1 h at 4 °C. Mitochondria were then separated using a magnetic separator column. The eluted mitochondria-enriched fraction was used for miRNA and protein analysis to evaluate the purity of the isolated mitochondrial fraction. Specifically, the non-mitomiR miR-370 was used as a negative control (Suppl. Fig. 2). The fractions for miRNA analysis were treated with RNAse A to remove extra-mitochondrial RNA, as described by Barrey et al. [12]. The reaction was stopped with proteinase K, then mitochondria were washed with the storage buffer and the pellets were stored at - 80°C until RNA extraction.

### Cell transfection

8×10^4^ cells were plated in 6-well plates and allowed to attach overnight before transfection with miR-34a, -146a and -181a miRVANA miRNA mimics (MC13089, MC10722, MC10421), miRVANA miRNA inhibitors (MH11030, MH10722, MH10421), miRVANA miRNA inhibitor negative control #1 (4464077) or with miRVANA miRNA mimic negative control #1 (4464058, all from Thermo Fisher Scientific, San Jose, CA, USA) at a concentration of 30 nM. Transient transfection was performed with TransIT-2020 transfection reagent (MIR 5404, Mirus Bio LLC, Madison, WI, USA), according to the manufacturer’s instructions. The ratio of transfection reagent (µl)/miR (µg) equal to 3:1 was found to be optimal. The transfection reagent–miR complex was prepared in serum-free medium. Analyses were performed 24 h after transfection.

### RNA isolation

Total RNA was isolated from HUVECs using the Norgen Biotek Kit (Thorold, ON, Canada), according to the manufacturer’s recommendations. RNA was stored at − 80°C until use.

### Quantitative RT-PCR of mature miRNAs

MiRNA expression was measured by qRT-PCR using the TaqMan miRNA assay (Catalog #4427012 Thermo Fisher Scientific). MiRNAs were reverse-transcribed with following manufacturer’s instructions (#4366596, Thermo Fisher Scientific). The qRT-PCR reaction mix included TaqMan MicroRNA assay, TaqMan Universal Master mix no UNG (4440040, Thermo Fisher Scientific) and RT product. The reaction presented an initial step at 95 °C for 2 min, followed by 40 cycles of 95 °C for 15 sec and 60 °C for 1 min. Data were analyzed with Rotor Gene Q (Qiagen, Hilden, Germany) with the automatic comparative threshold (Ct) setting for adapting the baseline. qRT-PCR data were standardized to RNU44. The 2^−ΔCT^ method was used to determine miRNA expression.

For mitomiR analysis from the isolated mitochondrial fraction, the ratio of 2^−CT^ mitochondrial miRs: 2^−CT^ cytoplasmic miRs was calculated in yHUVECs and sHUVECs. Data were normalized for cell number.

### Quantitative RT-PCR for mRNA expression

Total RNA (1 μg) was reverse-transcribed with QuantiTect Reverse Transcription Kit (Cat No./ID: 205311, Qiagen) according to the manufacturer's instructions. qPCR reactions were conducted on Rotor Gene Q 5plex HRM (Qiagen) in a 10 μl total reaction volume using SYBR Green JumpStart Taq ReadyMix (S4438, Sigma-Aldrich). The mRNA expression of the genes of interest was calculated with reference to two genes, β-actin and GAPDH. Each reaction was run in duplicate and always included a no-template control. The qRT-PCR reaction was programmed to start with a 2 min denaturation step at 95 °C for polymerase activation followed by 40 cycles of 10 sec denaturation at 95 °C, 30 sec of annealing at 72 °C, and 30 sec extension at 60 °C, during which fluorescence was measured. A melting curve was constructed by raising the temperature from 55 °C to 95 °C in sequential 0.5 °C steps for 6 sec. mRNA was assessed using the 2^−ΔCT^ method.

Primer sequences (5’-3’) were as follows: BCL-2 Fw: GGG GTC ATG TGT GTG TGG AGA G, RVCAT CCC AGC CTC CGT TAT CC; BCL-XL Fw: GGC CAC TTA CCT GAA TGA CC, Rv: AAG AGT GAG CCC AGC AGA AC; β-actin Fw: AAC TGG AAC GGT GGT CAA GGT GAC, Rv: CAA GGG ACT TCC TGT AAC AAT GC; GAPDH Fw: CCC TTC ATT GAC CTC AAC TAC ATG, Rv: TGG GAT TTC CAT TGA TGA CAA C.

### Immunofluorescent staining

HUVECs were seeded in EGM-2 media at a density of 1 × 10^4^ cells/well in BD Falcon chamber slide (cod. 354104). Cells were washed with PBS and fixed in 4% paraformaldehyde in PBS for 15 min at room temperature. Cells were washed again in PBS and incubated with 0.5% Triton X-100 in PBS for 5 minutes, blocked with 5% BSA for 1 hour at room temperature, followed by incubation with isoform B (LC-3B) antibody (cat. no. NB600-1384, Novus Biologicals, LLC, Littleton, CO, USA) in 1% BSA overnight at 4°C and with secondary anti-rabbit Alexa Fluor 488 antibody (cat. no. 111-545-003, Jackson Laboratories, Baltimore Pike, West Grove, PA, USA), at room temperature in 1% BSA for 1 hour. Cells were stained with nuclear HOECHST 33342 (cat. no. H-3570; Molecular Probes, Oregon, USA) in PBS for 5 minutes. Finally, cells were coverslipped with Vectashield mounting media (H-1200, Vector Laboratories, Burlingame, CA) and viewed with fluorescence microscopy (Nikon Eclipse 80i, Nikon, Japan). Omission of the primary antibody resulted in lack of labeling, confirming the specificity of the antibody.

### Protein extraction and immunoblotting

Total proteins were extracted using RIPA buffer (150 mM NaCl, 10 mM Tris, pH 7.2, 0.1% SDS, 1.0% Triton X-100, 5 mM EDTA, pH 8.0) containing a protease inhibitor cocktail (Roche Applied Science, Indianapolis, IN, USA). Protein concentration was determined using Bradford Reagent (Sigma-Aldrich, Milano, Italy). Total protein extracts (40 μg) were separated by SDS–PAGE and transferred to nitrocellulose membranes (Whatman, Dassel, Germany). Membranes were blocked in Tris-buffered saline with 0.1% Tween 20 (TBS-T) containing 5% fat-free dry milk and then incubated with anti-Bcl-2 (#2870, Cell Signaling Technologies, Danvers, MA, USA), anti-Survivin (NB500-201; Novus Biologicals, Milano, Italy), anti-p16(Ink4a) (sc-377412, Santa Cruz Biotechnology, Santa Cruz, CA, USA), anti-LC3 isoform B (NB600-1384, Novus Biologicals, LLC, Littleton, CO, USA), anti-VDAC (#4866, Cell Signaling Technologies, Danvers, MA, USA), anti-Lamin A/C (#2032, Cell Signaling Technologies, Danvers, MA, USA), and anti-β-actin (Santa Cruz Biotechnology, Santa Cruz, CA, USA) antibodies. Proteins were visualized by ECL (Amersham, Piscataway, NJ, USA) according to the manufacturer’s instructions and quantified using Quantity One software (Bio-Rad, Hercules, CA, USA).

### Cytokine production

Culture supernatants were collected at the end of each incubation, centrifuged, and stored at −20 °C until use in the assays. IL-1β concentrations were measured using a high-sensitivity commercial ELISA kit (Invitrogen, Thermo Fisher Scientific, Carlsbad, MA USA).

### Flow cytometry analysis

Intracellular ROS levels, mitochondrial functionality, apoptosis, and caspase activity were assessed by flow cytometry using special probes and a Guava EasyCyte flow cytometer with GuavaSoft 2.7 and an excitation source at 488 nm (Merck Millipore, Darmstadt, Germany). Emission fluorescence intensities were recorded in different channels on an average of 5,000 cells from each sample. Each condition was analyzed in triplicate and each sample was repeated twice.

### *Intracellular ROS assay*

ROS levels were evaluated by carboxy-2,7-dichlorofluorescein diacetate (carboxy-H_2_DCFDA) (Invitrogen). Cells were incubated with the dye (10 µM in PBS) in the dark for 30 min at 37 °C, washed and detached. An aliquot of each sample was then added to a solution of Guava Via-count (Merck Millipore), a fluorescent stain formulation that discriminates live from dead cells. Therefore, the analysis of ROS and viability were conducted simultaneously on the flow cytometer. Only viable cells were counterstained with Via-count to measure intracellular ROS, because carboxy-H_2_DCFDA can diffuse from cells with compromised plasma membrane, leading to false negative results. For data analysis, two “high ROS” regions were arbitrarily defined in the green channel using the fluorescence distribution of young HUVECs as a reference. The same gates were used to examine sHUVECs.

### *Mitochondrial superoxide anion assay*

Mitochondrial superoxide anion generation was assayed using MitoSOX™ Red (M36008, Invitrogen). Cells were stained following the manufacturer’s protocol. Briefly, MitoSOX was added to confluent cells and incubated at 37 °C for 10 min in the dark; cells were detached and analyzed by recording the fluorescence emission from the yellow channel. For quantitative analysis of distribution, gates defining regions with “low” and “high” superoxide anion levels were arbitrarily set using yHUVECs as a reference. The same values were used in all subsequent experiments.

### *Permeability transition pore opening assay*

mPTP opening was evaluated using MitoProbe^TM^ Transition Pore Assay Kit (Invitrogen) based on the manufacturer’s protocol. The assay is based on the calcein quenching method as described by Petronilli et al. [58]. Acetoxymethyl ester of calcein (Calcein AM) is a non-fluorescent dye that diffuses to all cellular compartments where intracellular esterases release the polar fluorescent dye calcein. Addition of cobalt chloride quenches cytosolic calcein, while mitochondrial calcein, which is not accessible to the quencher unless the mitochondrial membrane is permeabilized, remains fluorescent. Fluorescence intensity can therefore be used as an indicator of mPTP opening. Adherent cells were loaded with 250 nM calcein AM in presence of 400 µM cobalt chloride for 15 min at 37 °C. They were then detached, washed twice, and the green fluorescence was quantified by flow cytometry. The gates for distribution analysis were arbitrarily set using cells treated with the ionophore ionomycin as a positive control for opening.

### *Annexin V assay*

Annexin V is a calcium-dependent phospholipid-binding protein that binds phosphatidylserine on the surface of apoptotic cells. Used in association with 7-aminoactinomycin (7-AAD), a cell impermeant DNA intercalator, it discriminates apoptotic cells from live and dead cells. Floating and attached cells were harvested and washed with PBS; 10^5^ cells were resuspended in 200 µl annexin-binding buffer containing 5 µl annexin V (4300-0320; Merck Millipore). After incubation in the dark for 15 min at 37 °C, cells were washed twice, added with 200 µl buffer and 5 µl 7-AAD (4000-0110; Merck Millipore), and analyzed by flow cytometry within 1 h. The fluorescence results from the two channels were analyzed together, and gates for live, dead, and apoptotic cells were set arbitrarily using yHUVECs as the reference. The same setting was used for all the other experimental conditions. The results were expressed as percentage of apoptotic cells out of total live cells. Susceptibility to apoptosis was expressed as fold change of apoptotic cells compared to the respective untreated cells.

### *Detection of caspase-3 and caspase 1*

Activation of casp-3 and casp-1 was assayed with FAM FLICA probes (ImmunoChemistry Technologies, Bloomington, MN, USA). FLICA is a cell permeant that binds covalently the active caspases and is revealed by a green fluorescent signal. Cells were stained following the manufacturer’s instructions. Briefly, cells were detached, washed, and stained with 1 x FLICA solution in the dark for 50 min at 37 °C, counterstained with propidium iodide, and immediately analyzed. The fluorescence intensity from the two channels was analyzed together.

### TEM analysis

Cells were plated on Aclar films (Ted Pella, Redding, CA, USA) for flat embedding. They were fixed for 1 h at room temperature with a solution of 2.5 glutaraldehyde in 0.1 M cacodylate buffer (pH 7.4), postfixed in 1% osmium tetroxide in 0.1 M cacodylate buffer for 30 min, dehydrated in an acetone series, and embedded in epoxy resin (#43359, Sigma, Milano. Italy). Ultrathin (40 nm) sections were stained with lead citrate and uranyl acetate and examined in a CM12 transmission electron microscope (Philips Nederland) at 100 kV**.** Images were digitally captured using an Olympus Veleta (Japan) or a Megaview G2 (Olympus Soft Imaging Solution Münster, Germany) digital camera previously calibrated for every magnification used. Calculation of mitochondrial solidity from young and senescent HUVECs was performed using the embedded features of the image processing software ImageJ (NIH Bethesda, USA). Mitochondria were manually selected on the original image and fed to the particle analysis module to measure solidity [59]. For a bi-dimensional image this parameter is calculated as the fraction of pixels contained in a convex polygon (fitted around a mitochondrion) which is also mitochondrial pixels. Low values (close to 0) are more often associated with tortuous mitochondria with a non-uniform shape, whereas high values (close to 1) are frequently associated with more uniformly shaped, compact mitochondria that do not show high levels of branching [29,59]. Semi-quantitative analysis of autophagic vesicles was obtained by counting them on each image representing unique cell or part of it and showed as number of vesicles x cell area.

### Statistical analysis

Data are presented as mean ± standard deviation (SD) of at least 3 independent experiments. Student’s t test was applied to determine differences between samples. P values < 0.05 were considered significant.

## Supplementary Material

Supplementary Figures
