# Supplementary material for: The mitomiR/Bcl-2 axis affects mitochondrial function and autophagic vacuole formation in senescent endothelial cells
Source: Aging (Albany NY). 2018 Oct 21;10(10):2855–73. doi: 10.18632/aging.101591 (PMC6224225; doi:10.18632/aging.101591)
Supplement: Supplementary Figures [file aging-10-101591-s001.pdf]

SUPPLEMENTARY FIGURES

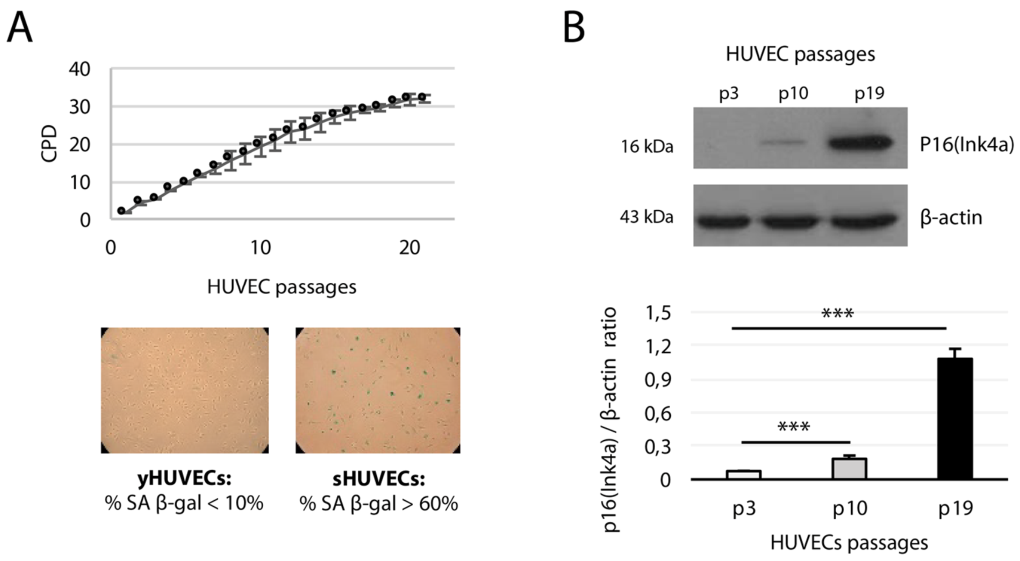

Supplementary Figure 1. Phenotypical characterization of HUVECs. (**A**) Growth curve showing cumulative population doublings (CPDs) of three different HUVEC pools undergoing replicative senescence (X axis: cell passages from P1 to P21) and representative positivity for SA β-Gal in young (< 10%) and senescent HUVECs (> 60%) cells. (**B**) Western blot and densitometric analysis of p16(Ink4a) at passages p3, p10, and p19 of the growth curve. Protein expression values are reported as p16(Ink4)/β-actin ratio.

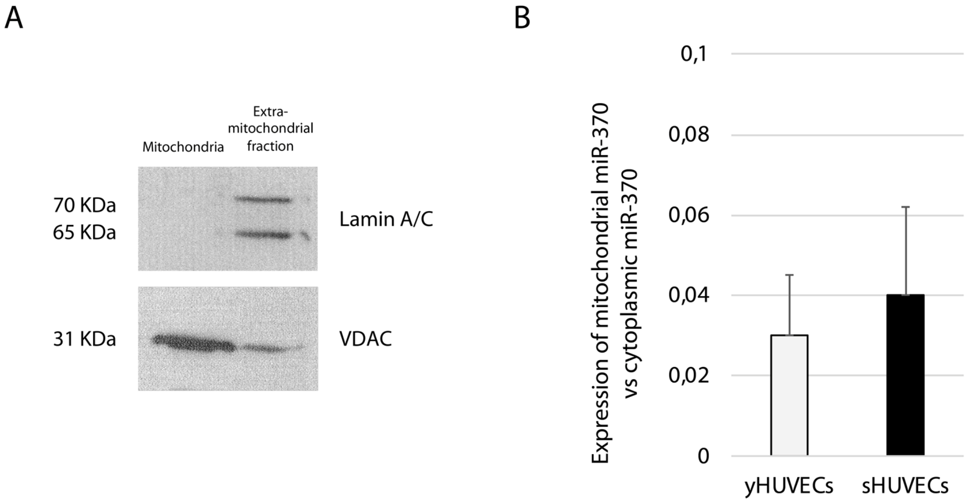

Supplementary Figure 2. Purity assessment of isolated mitochondria. (**A**) Western blot analysis of Lamin A/C and VDAC from mitochondria-enriched and extra-mitochondrial (cytosol and nuclei) fractions after mitochondria isolation. (**B**) Ratio of miR-370 expression in the isolated mitochondrial fraction to the cytoplasmic fraction in yHUVECs and sHUVECs.
